# Reply to Mehrotra et al. Wastewater-Based Surveillance Does Not Belong in a Regulatory Framework Designed to Protect Waters That Receive Treated Wastewater. Comment on “Wright, T.; Adhikari, A. Utilizing a National Wastewater Monitoring Program to Address the U.S. Opioid Epidemic: A Focus on Metro Atlanta, Georgia. *Int. J. Environ. Res. Public Health* 2023, *20*, 5282”

**DOI:** 10.3390/ijerph20176637

**Published:** 2023-08-24

**Authors:** Tamara Wright, Atin Adhikari

**Affiliations:** 1 University College, University of Denver, 2211 South Josephine Street, Denver, CO 80208, USA; 2 Jiann-Ping Hsu College of Public Health, Georgia Southern University, 501 Forest Drive, Statesboro, GA 30460, USA; aadhikari@georgiasouthern.edu

We are honored that authors are reading our manuscript [1], and that we have sparked compelling conversations among water professionals, researchers, and people within academia. After reading the comment paper written by Mehrotra et al. [2] titled “Wastewater-based surveillance does not belong in a regulatory framework designed to protect waters that receive treated wastewater”, we wanted to address the controversial concerns that were mentioned. We also would like to reiterate, with our novel approach [1], that water professionals and public health professionals have the opportunity to collaborate on combatting the opioid epidemic, while, simultaneously, protecting aquatic ecosystems from opioid discharges.

As the opioid epidemic continues to impact public health globally and within the United States, more researchers are suggesting and emphasizing the importance of utilizing a national framework and a multi-faceted, integrated approach to tackle the opioid epidemic [3,4]. As we stated in our manuscript, the National Wastewater Surveillance System (NWSS), which was established by the Centers for Disease Control and Prevention (CDC) and other public health professionals [5], would be a great tool to utilize as a national framework to tackle the opioid epidemic without changing the current operating framework. The NWSS would not require wastewater treatment plants (WWTPs) to remove opioid metabolites, which inherently could increase their budget; hence, this is the reason the National Pollutant Discharge Elimination System (NPDES) would be a more effective, synergistic approach that can be integrated as a national framework [6]. The detrimental harm to ecosystems from opioid metabolites released through treated wastewater is a challenging issue, and that problem should be separately considered with our proposed approach.

Additionally, as we mentioned within our manuscript, several countries have utilized wastewater surveillance in their national frameworks to monitor their respective opioid epidemics [7,8,9,10,11,12]. These national programs have yielded great successes in collaborating with wastewater operators and other employees at WWTPs on collecting composite samples and conducting laboratory analyses to assess opioid metabolites in wastewater [7,8,9,10,11,12]. Due to the success of the opioid monitoring process within wastewater, these countries have been able to provide a significant amount of resources (e.g., more intervention centers, policies, harm reduction techniques) to address the opioid epidemic within their communities. However, without a national wastewater monitoring framework that assesses opioids, the success in addressing the opioid epidemic within their communities would be significantly delayed.

Our original manuscript [1] did not provide a detailed discussion on the impact that opioids have on aquatic ecosystems because that was not the focus of the paper. We wanted to focus on surveillance of the opioid epidemic by using an alternative novel approach involving the NPDES. We have found several articles that mention the impact that opioids have on aquatic life. Mehrotra et al. [2] mentioned that “the reported concentrations of opioids and their metabolites in treated effluent are usually less than 1 μg/L, and concentrations in receiving waters are likely to be lower, depending on concentrations in the water body upstream of the discharge” [2]. We disagree with these claims. Researchers have determined that illicit drugs may remain in untreated effluents, being transferred in the final effluent into aquatic environments (groundwaters, rivers, ocean), affecting water quality and ecosystem health [13,14]. Fontes et al. [15] has provided several tables within a published article that provide information on the amount of opioid metabolites that have been discovered in influent composite samples, and the amount of opioid metabolites that have remained within the effluent discharges after the wastewater treatment process. The research presented within this article has been conducted within WWTPs across the globe [15]. Within Table 1, we have shown some of these opioid metabolites that remained in effluent discharges in accordance with the research collected by Fontes and other researchers [15]. Researchers have also monitored waterways within the United States and determined that high concentrations of opioid metabolites (e.g., cocaine, THC, amphetamines, and MDMA) were present within various surface waters within various cities throughout the United States [16,17,18]. More studies within the United States are necessary to provide a more thorough analysis of the opioid metabolites that are present within U.S. waterways.

Moreover, Taylor [31] mentions how opioids have a significant impact on marine health by causing animals and fish who consume opioids within their habitat to become addicted. Researchers from the Puget Sound Institute have been monitoring and analyzing opioid exposure in mussels within the Puget Sound, which is an inlet that leads into the Pacific Ocean [32]. Recently, the researchers discovered that trace amounts of oxycodone, antibiotics, antidepressants, chemotherapy drugs, and heart medications L-an abundance of medications that are found within the Puget Sound are attributed to the effluent from wastewater treatment plants, where opioid metabolites are not monitored and remain untreated [32].

In addition, significant research has been conducted to assess the impact that illicit drugs have on aquatic ecosystems. More research is necessary to determine the acute and chronic impact that each opioid metabolite has on the specific species within the aquatic ecosystem [15]. The impact that cocaine has on aquatic ecosystems has been thoroughly evaluated by several researchers. Souza et al. [33] assessed the toxicity of cocaine combined with different scenarios of ocean acidification. It was determined that when an area had a high concentration of cocaine combined with ocean acidification (6.25; 12.5; and 25 mg L^−1^) and the pH value decreased from the neutral pH range (7.5 and 7.0), the development of the embryo larva of the *Echinometra lucunter* sea urchin was negatively impacted [33]. Other researchers [34] determined that when zebrafish embryos were exposed to cocaine (20 ng·L^−1^), their cell viability reduced significantly, DNA fragmentation increased, and there was an onset of apoptotic cells and micronuclei. Furthermore, researchers [35,36] have discovered that high concentrations of cocaine significantly impact the endocrine system of eels because exposure to cocaine metabolites increases brain dopamine and plasma catecholamines, and affects their brain, muscle, and liver which also contributes to their decline, and therefore contributes to the adverse effects that contaminated fish consumption can have on human health.

Once opioid metabolites are excreted in the sewage, the substances are transported to the WWTPs. Several authors have mentioned that most WWTPs are not designed to remove opioid metabolites, which results in contaminated effluents being discharged into waterways [15,37,38,39,40,41,42]. Recent studies have shown that illicit drugs may present risks to human health and the environment, since they can interact biologically with non-target organisms [43,44]. If opioid metabolites were placed on NPDES permits, WWTPs would have to implement and utilize more innovative approaches to remove opioid metabolites from the wastewater treatment process. More research is necessary to evaluate which wastewater treatment processes are most effective in preventing opioid discharges from impacting the waterways.

Mehrotra et al. [2] emphasized the need to separate wastewater-based epidemiology and the NPDES in regard to protecting waterways. Mehrotra et al. [2] stated that “NPDES permit limits are developed to meet water quality standards to protect human health and aquatic life in water bodies that receive pollutants discharged from point sources”, and the authors believe that there is not enough research to “support the conclusion that human health or aquatic life is at risk from opioid compounds and their metabolites in waters impacted by wastewater effluent” [2]. We dispute these claims because there is a substantial amount of research that emphasizes the detrimental impact opioid metabolites have on aquatic life, and the importance of removing opioid metabolites from the wastewater treatment process [15,16,17,18,19,20,21,22,23,24,25,26,27,28,29,30,31,32,33,34,35,36,37,38,39,40,41,42,43,44].

We understand that adding opioid metabolites to the NPDES is a novel approach, and we agree that “adding water quality standards for new compounds, such as opioids, would need to follow the deliberate process that has been designed to incorporate the most credible scientific evidence on risks and ensure that new water quality objectives are implemented where appropriate” [2]. We also agree with Mehrotra et al. [2] and have mentioned within our article that the economic feasibility of implementing this approach may be difficult as it can be expensive [1]. As we have seen with the ongoing efforts centered around per- and polyfluoroalkyl (PFAS) regulations in drinking water and wastewater, and the Bipartisan Infrastructure Law, when an emerging issue is impacting the nation, funding can be provided to assist communities with the assessment and monitoring of water contaminants and pollutants. Mehrotra et al. [2] mentioned that the laboratory analysis (either on-site or through a contract lab) would be expensive; but, if the wastewater professionals are not provided funding and training on how to conduct the proper analyses, then collaborating with public health professionals and toxicologists, who have the experience on how to conduct these analyses, would be ideal.

To elaborate on the execution of this approach, opioid monitoring through the NPDES could be an assistance tool within the most impacted communities. The Environmental Protection Agency (EPA) gives primacy to the states, which allows the state’s environmental departments to determine which water quality parameters each wastewater treatment plant has to adhere to, in accordance with the NPDES permit [6]. The water quality parameters on the NPDES permit could include the treatment and removal of pH, total suspended solids (TSS), biochemical oxygen demand (BOD), ammonia, total phosphorus, oil and grease, turbidity, dissolved oxygen, temperature, and fecal coliforms, as well as opioid metabolites [1]. We are not proposing that each NPDES permit throughout the nation requires wastewater treatment professionals to monitor opioid discharges (e.g., rural communities who lack adequate resources that are not impacted by the opioid epidemic), as mentioned by Mehrotra et al. [2]. We are proposing that if there is an increase in opioid consumption in a given area, and there is collaboration between public health professionals and state environmental professionals, then the use of the NPDES permit is an effective tool that can monitor the emerging crisis within this area.

As the opioid epidemic continues to prevail and have a detrimental impact on public health, more researchers are suggesting and emphasizing the importance of utilizing a national framework to tackle the opioid epidemic [3,4,15]. The consumption of illicit drugs worldwide continues to be a relevant social problem, but it is also being recognized as an environmental concern due to the vast amount of opioid metabolites that have been discovered within waterways (e.g., wastewater, freshwater, drinking water, groundwater, and seawater) [15]. Researchers have also demonstrated the fragility of wastewater treatment systems due to the lack of illicit drug and opioid metabolite removal from effluents, and the occurrence of these opioid metabolites within aquatic ecosystems [15]. Furthermore, with the occurrence of opioid metabolites within effluents, acute and chronic impacts are present within aquatic ecosystems; these impacts should be addressed by academics, environmentalists, and policymakers to improve viability among aquatic ecosystems [15].

In regard to the opioid epidemic that is occurring within the United States, there is still a need for a national approach that can operate continuously in order to tackle the opioid epidemic and reduce its impacts on aquatic ecosystems. The national framework should utilize collaborative efforts across multiple systems to coordinate comprehensive data and generate predictive models of multiple communities to determine the impacts and accessibility of the necessary resources (e.g., interventions, policy, and enforcement) [3]. We continue to emphasize the importance of bridging the gap between water professionals and public health professionals in order to live in a sustainable world, where aquatic ecosystems are preserved, and longevity and viability are increased among humans; it is necessary to utilize multiple approaches to achieve this goal. Working professionals (i.e., public health professionals and water treatment professionals) should not be required to solve societal and environmental problems unilaterally when a collaborative effort is imperative and plausible.

## Figures and Tables

**Table 1:** Range of influent and effluent opioid compound concentrations reported worldwide in wastewater treatment plants.

Compound	Country	Influent Concentration (ng·L^−1^)	Effluent Concentration (ng·L^−1^)	Reference(s)
Morphine	Spain	<LOQ–96.7 ^1^	<LOQ–81.1 ^1^	[19]
	Switzerland	<LOQ–1970 ^1^	84.0–1270.0	[20]
	England	65.6–985.5	13.0–266.6	[21]
	Spain	90.0–275.0	60.0–155.0	[22]
Cocaine	England	5.10–208.90	0.6–70.3	[21]
	United Kingdom	109.0	65.2	[23]
	France	4.80–282.0	<LOQ–20.7 ^1^	[24]
	USA	465.0–1030.0	<10	[17]
Codeine	Netherlands	240.0–536.0	173.0–245.0	[25]
	Germany	540.0	260.0	[26]
	Italy	275.0–335.0	110.0–126.0	[27]
	United Kingdom	2703.50	1206.20	[23]
MDMA	Croatia	12.0–160.0	8.80–81.0	[28]
	Netherlands	<12.0–140.0	30.0–138.0	[25]
	Spain	2.0–598.0	2.0–267.0	[29]
	United Kingdom	137.90	155.70	[23]
Methadone	Spain	4.0–23.90	4.0–24.70	[19]
	Switzerland	42.0–202.0	44.0–128.0	[20]
	United Kingdom	171.10	68.8	[23]
	England	2.60–171.10	1.40–91.0	[21]
Methamphetamine	China	42.20–447.20	0.6–112.0	[30]
	USA	255.0–355.0	<25.0	[17]
	England	17.4–3112.5	4.30–145.20	[21]
	Spain	212.0–1021.0	215.0–325.0	[22]

^1^ Limit of Quantification (LOQ) indicates lowest concentration at which analyte is detected.

## Data Availability

Publicly available datasets were utilized within this manuscript. The data can be found within the following article: https://doi.org/10.1007/s11356-020-08375-2 [15].
